# Considerations for Clinician-Educators Developing Online Educational Content: A Narrative Review

**DOI:** 10.7759/cureus.55048

**Published:** 2024-02-27

**Authors:** Amiran Baduashvili, Brandon Fainstad, Elizabeth L Kudron

**Affiliations:** 1 Department of Medicine, University of Colorado Anschutz Medical Campus, Aurora, USA; 2 Department of General Internal Medicine, Veterans Administration Eastern Colorado Health Care System, Aurora, USA; 3 Department of Pediatrics, University of Colorado Anschutz Medical Campus, Aurora, USA; 4 Department of Biomedical Informatics, University of Colorado Anschutz Medical Campus, Aurora, USA

**Keywords:** digital education, elearning, accessibility, cognitive load, copyright, intellectual property rights, online curriculum development, online content development, medical education

## Abstract

In modern medical education, clinician-educators are increasingly called upon to develop online education to complement or replace in-person instruction. Despite a growing need for online curricula, many medical professionals lack training and experience in digital content development, deployment, assessment, and maintenance. Previous studies offer guidance on some aspects of online education development but often overlook key components, such as accessibility, legal considerations, financial implications, and sustainability challenges. This review offers medical professionals a broad overview of these important issues. We discuss various pedagogical considerations, including aligning educational goals and objectives with the digital content, choosing the appropriate online interface, and employing strategies to mitigate cognitive load while maximizing accessibility to create an inclusive online learning environment. We offer practical tips for creating effective, high-quality, and enduring audio-visual content and reflect on initial content deployment, testing, assessment, and revision. We discuss the intricacies of obtaining continuing medical education credits when the target audience includes faculty members. We address several legal issues online educators must consider, such as copyright laws, intellectual property rights, and medical liability. The review concludes with a discussion of sustainability mechanisms and financial considerations to ensure the long-term success of the educational program. Our recommendations aim to equip medical professionals embarking on a digital education journey with practical tools to produce effective, inclusive, and sustainable online content while considering legal implications.

## Introduction and background

The COVID-19 pandemic has accelerated a transition from traditional in-person teaching to online education [1-7]. Clinician-educators may find themselves leading virtual didactics and creating online materials for both synchronous and asynchronous learning [3,8,9]. Synchronous education refers to live teaching, where the student and teacher are in the same online environment, whereas asynchronous learning refers to the student engaging with learning materials in a self-paced manner without the presence of an instructor. A recent comprehensive literature review by Bankar et al. demonstrated that various online modalities have been utilized in medical education, including videos, online quizzes, simulation, and virtual reality [10]. The review highlighted multiple benefits of online education, including ease of access, cost-effectiveness, utilization of interactive and personalized learning, and potential for improved sustainability with the reduced need for in-person presence and classroom infrastructure.

The review also noted several limitations of digital education, such as inconsistent quality control and inadequate teacher training in online education delivery [10]. Indeed, many educators need more experience and expertise in online course design and content creation [11-13]. A study evaluating 26 faculty members at a large academic institution in the United States (US) found that many of them felt inhibited by technological challenges, unfamiliarity with the needed resources, and concerns about legal implications, such as violating copyright laws [14]. Recent publications have addressed specific areas of online content development, ranging from needs assessment, implementation, and evaluation [15] to the creation of effective educational videos [16]. However, to the best of our knowledge, reviews to date have yet to address the broad range of considerations for online educators, from the technical aspects to the legal implications.

Herein, we explore methods to create high-quality digital content and present it to users in ways that limit their cognitive load and expand accessibility. We discuss the financial and legal considerations of online curricula, including continuing medical education (CME) accreditation, copyright laws, and intellectual property (IP) rights, primarily from the perspective of the US legal landscape. We do not explore the dissemination or marketing of online curricula. We mainly focus on asynchronous online learning materials such as podcasts, modules, and videos, but many of the concepts outlined here are also relevant to synchronous online teaching.

## Review

Initial considerations

The content of online education can range from quick reference guides or standalone videos to comprehensive platforms with longitudinal user engagement. Several factors should be considered when deciding on the most appropriate digital medium, format, and platform for presenting online education. These include the goals and scope of the content, the target audience, learner assessments, and available funding and resources, among others.

Evidence suggests there may be racial, ethnic, gender, and age-based differences in the preferred online education type and format [5,17]. Thus, educators may adjust their educational methods based on the target audience's preferences. For example, previous research on online education has shown that female students may prefer asynchronous courses, while Black students may prefer synchronous sessions [5].

While established methods [18] for content creation can inform curriculum development, these recommendations typically need to be adapted in the online setting [15]. Creators should consider how the digital environment may impact curricular goals and objectives. For instance, a goal for learners to improve their motivational interviewing skills may have previously been achieved through in-person role-playing. The question then arises: can such a goal be achieved in an online environment, or will the online content mainly serve to augment the in-person experience? Less comprehensive online content may suffice if the latter objective is the case.

When teaching online, creators need to consider how the teaching topic can be presented most effectively to promote engagement, understanding, and retention. A variety of digital formats are available, including video recordings, interactive slides, embedded quizzing, and live online discussions. It is important to select a format or mix of formats that conveys the subject with most clarity and efficiency. For procedural instruction, a video or live demonstration would be preferable, while slides can be used to outline steps and present safety checklists. Embedded quizzes can reinforce the steps and checklists and assess for retention.

Creators should also consider if and how they plan to evaluate the developed content or assess learners’ performance. If neither aim needs to be attained, posting materials on commonly used free platforms and sharing them with learners may be adequate. If users will be accessing the content as a quick reference on their mobile phones, ease of access and mobile-friendly navigation are critical features to consider. Course feedback can be gathered through integrated or asynchronous survey instruments. Alternatively, advanced platforms such as learning management systems (LMS) may be necessary to provide user authentication, confidentiality, progress tracking, assessment, and direct feedback, among other desired curricular functions.

Cognitive load

Online education offers students flexibility for scheduling and pacing their learning when delivered asynchronously. However, the online environment can potentially increase the learner’s cognitive load through additional extraneous load (EL), input that is irrelevant to the learning [19]. The handling of online devices and the noise generated from the online environment contribute to excess EL [20]. By minimizing EL, educators will enhance learner engagement and long-term knowledge retention [21].

Some techniques [22] that will reduce cognitive load include clarifying the purpose and scope through an introductory pre-lecture and learning objectives; ensuring simple navigation with an interactive table of contents; and information chunking, or grouping large amounts of material into smaller parts. Videos should be segmented with linked bookmarks to allow quicker access to relevant content in a more extensive video. The text should have a plain background with adequate font size, headings, and color coding. For example, when teaching diagnostic test characteristics, the creators may consider designating and consistently using a specific color for each critical variable, such as sensitivity, specificity, and likelihood ratios. Longer or less relevant supportive information should be separated into linked appendices.

Accessibility

There are several considerations to maximize accessibility for persons with sensory disabilities or other limitations. Image labeling provides a readable substitute for those with visual impairment or poor internet connection that may interfere with image viewing. Appropriate contrast between text and background color can further improve the experience of those with limitations in color perception. The recommended contrast ratio [23] between background and text is 4.5 to 1 for small and 3 to 1 for large texts. Background-to-text contrast-checking tools are freely available online (i.e., www.webaim.org).

Closed captions can be helpful for those with hearing impairment or limited language proficiency. Some video editing software and hosting platforms provide options for automatically generated closed captions and transcriptions. The content creators can usually edit these to ensure correct transcription and spelling. Creators should avoid using high-frequency flashing lights in their videos (more than three flashes per second) or provide adequate warnings for those with seizure predispositions [23].

Creating video

With advances in smartphone hardware and internal processing power, it is possible to seamlessly record, edit, and publish content from the same device. While the ease of this approach should not be overlooked, there remain considerable advantages to transferring video content to a computer where more sophisticated programs offer additional capabilities with splicing, voice-over, annotations, and graphics. The visual content will frequently involve tables, figures, images, drawings, or text that the educator manipulates and annotates as the video progresses. Educators may consider several features when choosing video-production and editing software: (1) availability of annotation and/or pointer tool; (2) ability to record video and audio separately; (3) ease of editing, including retrospectively changing text color or video background, correcting spelling errors, or removing filler words/sounds; and (4) ability to export video files into accessible formats. The flexibility to record audio and video separately may help an educator focus on word choice and avoid recording extraneous sounds generated while concurrently drawing or annotating. Highly customizable video-editing software can allow educators to easily incorporate learner feedback, edit errors, or update the recording for future iterations without re-recording the entire session. Additional considerations for creating effective educational videos have been previously published [16].

Educators recording synchronous sessions may need to consider other issues. As adequate lighting can be challenging to accomplish, it is essential to reduce the background and overhead lighting and maximize the lighting facing the educator in a video recording. Whenever a tablet or phone is used for recording, it should be positioned at eye level in landscape orientation rather than portrait to ensure better compatibility with large-screen devices.

High-quality audio

High-quality audio will enhance the overall learner experience and can be even more crucial than video quality. Numerous hardwired or Bluetooth-accessible devices provide acceptable quality. Additional steps and equipment may be necessary if the goal is to record exceptional audio. Creators should consider practicing content delivery prior to recording, with special attention to the enunciation of words and the optimal distance from the recording device needed to achieve ideal sound quality while avoiding breath sounds being recorded. Educators should be attuned to ambient noise (central air, outside traffic, construction noise, etc.) and echoes, as these sounds could be recorded and distract learners. Some institutions may provide access to sound-proof studios. If recording in a fully quiet environment is not possible, post-recording sound processing features that remove ambient noise can be utilized through video editing software.

Content creators may encounter two types of microphones: USB (digital) and XLR (analog). USB microphones plug directly into a computer without additional hardware. XLR microphones require extra hardware, such as an audio interface, to convert the analog signal to digital. Although many USB microphones provide good recording quality, XLR microphones are an excellent option for achieving professional-quality audio recording. Further refinement of audio quality can be achieved by positioning a pop filter between the speaker and the microphone, attenuating artifacts produced by enunciating certain letters like “P’s” and “B’s.” Creators should consider scripting dialogue to minimize filler words, extraneous sounds, and delays.

Selecting digital interface

Several factors may influence the decision of which user interface and platform are best suited for hosting developed educational content. The two primary considerations are the type of teaching materials and how these are intended to be interacted with. Free and widely available video platforms may be appropriate if the course primarily consists of video instruction. The videos can be de-listed, so they are only accessible to those with a private web link. Comment sections can be utilized as discussion boards. Advanced features may allow adding quizzes within a video to make the content more interactive.

LMSs provide additional features such as integrating case vignettes, learner assignments, user tracking, discussion boards, systematic feedback collection, and advanced testing features. Educators should consider using LMSs to create assessments. These platforms provide flexibility in test design and store responses directly into a secure database that may be accessed for detailed analysis. Several LMS options are available, some with free basic versions. Educators may have free access to one or more of these LMS providers through their institutions.

Testing and revision

Evaluating developed content with a suitable audience and revising materials to achieve intended objectives is essential [15]. Depending on the online platform, educators may consider pilot testing to ensure ease of access and navigation of course content. There are several considerations that can be customized to fit specific educational needs. Pilot test content on various devices and explore compatibility with different web browsers. If a compatibility issue is noted, provide an adequate warning during content distribution. For complex online courses with multiple modules and interactive elements, near-live pilot testing with a sample target audience allows for directly observed experience and feedback from the relevant user. For instance, accessing the course may require reading a dissemination email, following a web link, logging into the host software, finding the right content, and navigating through it. To ensure a seamless experience, the course director may schedule recorded video calls with a few volunteer users, watch them follow the instructions to access the course, and ask them to think aloud through their experience. Appropriate changes should be made if significant issues are identified, and testing should be repeated until the course and instructions are clear and user-friendly. To address unanticipated problems, educators may also consider conducting semi-structured qualitative interviews with initial course users to gather valuable insights before making materials accessible to a broader audience.

CME credits

In the US, medical licensing organizations and institutions may require clinicians to earn CME credits. Providing CME credits can help incentivize faculty learners to engage with online courses. However, CME accreditation can be costly and complicated. The Accreditation Council of Continuing Medical Education (ACCME) provides accreditation to organizations as CME providers. In addition to dispensing CME, these CME-provider organizations may award CME credits to individual courses or educational activities. Educators seeking CME accreditation for an online course may appeal to their local CME-provider accredited institutions. Individual organizations, not ACCME, set the cost per CME credit that an educator incurs. Therefore, CME accreditation costs vary; some local institutions may not provide the most competitive rates. Educators should explore CME accreditation options broadly and consider professional medical organizations as alternative venues for accreditation. Costs may vary by content type, such as a single live event, recurring event, or enduring material (an online course). Typically, enduring materials and recurrent events are more costly and incur annual CME fees. Creators should consider such costs when constructing a sustainability model for their educational content.

Copyright

Historically, US educators have been protected by Fair Use exceptions that allow them to use external copyrighted material for teaching [24]. However, these exceptions mainly apply to in-class instruction [25,26]. Distance learning allows for a wide distribution of copyrighted material and a potential for monetization of the digital content; thus, additional steps may be necessary to ensure compliance [25,27]. Terms of use policies are typically located at the bottom of a webpage for the publishing entity. Copyrighted materials have the most restrictive terms of use, establishing that the individual or organization owns the content and may not be shared, reproduced, or modified without permission. Copyright protection exists simply by creating content and including a visible copyright notice. It is not necessary to register the copyright. However, content owners can communicate terms of use through a licensing agreement that explains how and if the content may be reproduced or adapted for commercial or non-commercial purposes.

Educators may be interested in using tables and figures from peer-reviewed or other publications. Permission may be requested by contacting the owner or by purchasing the copyright. Most peer-reviewed journals provide links to relevant copyright resource pages where rights to use or reproduce content can be accessed or purchased. Alternatively, the publisher may provide more liberal Terms of Use, most popularly done through a Creative Commons license [28], which requires the work to be attributed to the licensor and provides one of six licensing combinations that clarify permissions for adaptation and commercialization. For example, an ‘Attribution-Non-Commercial 4.0’ or CC BY-NC 4.0 will require attribution and allow the work to be copied, adapted, and re-distributed, but not for commercial purposes.

Intellectual property rights

Educators who create online content may ponder who owns the content, especially if they move to a different institution or if the content has the potential to be monetized. The US Copyright Revision Act of 1976 assigns content ownership to the creator. However, the ‘work for hire’ doctrine provides a major exception. If the creator is employed and the work is produced within the scope of employment, the employer assumes ownership rights [26,29]. Using institutional equipment, funds, or paid time to develop course content may strengthen the ‘scope of employment’ argument for institutions seeking to claim the content’s IP rights.

Prior versions of the law provided ‘textbook exception,’ which assigned the instructor the rights to course syllabi, textbooks, or other instructional materials. That exception was omitted from the 1976 revision. Institutions have continued to honor the exception, but such exception is not codified in law, and employers can claim IP rights to content produced by faculty [26]. Given lower overhead costs and a greater audience, the potential profits from online courses are higher, which can create financial and legal tension [29,30], changing the pre-existing landscape. Some but not all institutions have established policies about IP that content creators can consult. Suppose the content creator anticipates the course will generate revenue for self-sustainability. In that case, they should consider contacting relevant institutional bodies and discussing potential content ownership and profit-sharing agreements before developing or disseminating the content.

Sustainability

Mechanisms for long-term funding are important to contemplate at the onset of a project. In medical education, the primary consideration is whether to seek long-term funding through an employer or external sources. There are advantages and disadvantages to either path.

If an institution is to provide long-term support, local resources can be leveraged to cover start-up costs and reduce expenses. The project could be initiated through an institutional grant. Costs can be avoided by accessing free or subsidized institutional library resources, image- and video-editing software, data-management systems, information technology support, and more. Creators may need to provide the sponsoring institution with compelling evidence for the added value or return on investment (ROI) of developed content to justify ongoing costs. The value can originate from improved education, which may lead to improved quality of care and reduced medical errors and associated costs. Academic institutions may generate ROI through publicity of a widely disseminated sponsored course. Alternatively, educators could appeal to the institution’s academic mission through research and scholarly output.

Long-term funding outside the institution requires considerable forethought from the creator to avoid conflict over IP rights. Creators will be on firmer legal ground if they only use institutional resources if prior written agreements between the two explicitly grant ownership to the individual and acknowledge the use of these resources.

Content may be sold for a one-time fee or a recurring subscription. An increasingly popular alternative to charging users is to make the content freely accessible and reproducible for commercial purposes. This method may generate revenue through advertising and sponsorship, which rely on traffic and exposure. Revenue may also be sought through donations and external grants, in which creators may appeal to contributors’ desire for freedom of information and improved access to high-quality education. In those cases, it may be favorable for the owners to have less restrictive content protection through a Creative Commons license.

Medical liability

In medical education, particularly for publicly available content, it is essential to avoid liabilities associated with formal medical advice, institutional representation, and protected patient health information. This can be addressed through a disclaimer made easily visible to students. Herein, we provide three sample statements to address common liability concerns. (1) “This content is intended for educational purposes only. This content is not intended, nor should it be used as medical advice.” (2) “The information presented here may represent the opinions of the authors but does not represent those of their employing institutions or medical societies in which they are involved.” (3) “The clinical information presented here does not represent any one individual. All uniquely identifiable data has been removed to protect the privacy and confidentiality of patients.” If the educational content functions outside of an institution's direct ownership and protection, it is advisable to further reduce liabilities through incorporation as a Limited Liability Corporation or similar entity.

## Conclusions

The increased demand for online learning provides new challenges for clinician-educators. This evolving landscape will push educators to develop novel skills to better serve current and future generations of learners. A variety of resources are available to produce high-quality audio and visual content. We recommend a mindful approach to online content creation, focusing on maximizing accessibility and decreasing cognitive load. Online course designers should be prepared to navigate legal and financial challenges to increase the success and sustainability of their online educational content.
